# A Novel Panel of Rabbit Monoclonal Antibodies and Their Diverse Applications Including Inhibition of *Clostridium perfringens* Epsilon Toxin Oligomerization

**DOI:** 10.3390/antib7040037

**Published:** 2018-10-25

**Authors:** Jennifer R. Linden, Kiel Telesford, Samantha Shetty, Paige Winokour, Sylvia Haigh, Ellen Cahir-McFarland, Giovanna Antognetti, Abhishek Datta, Tao Wang, Werner Meier, Timothy Vartanian

**Affiliations:** 1 Brain and Mind Research Institute, Weill Cornell Medical College of Cornell University, New York, NY, 10065, USA; jel2049@med.cornell.edu (J.R.L.); kit2003@med.cornell.edu (K.T.); samanthashetty25@gmail.com (S.S.); pwinokur@mail.rockefeller.edu (P.W.); sylviavhaigh@gmail.com (S.H.); 2 Rockefeller University, New York, NY 10065, USA; 3 Biogen, Cambridge, MA 02142, USA; ellen.cahir-mcfarland@biogen.com (E.C.-M.); giovanna.antognetti@biogen.com (G.A.); dradatta2004@gmail.com (A.D.); wt76610@gmail.com (T.W.); werner.meier@biogenidec.com (W.M.)

**Keywords:** *Clostridium perfringens*, epsilon toxin, epsilon protoxin, neutralizing, antibodies, oligomerization, pore formation

## Abstract

The pore-forming epsilon toxin (ETX) produced by *Clostridium perfringens* is among the most lethal bacterial toxins known. Sensitive antibody-based reagents are needed to detect toxin, distinguish mechanisms of cell death, and prevent ETX toxicity. Using B-cell immuno-panning and cloning techniques, seven ETX-specific monoclonal antibodies were generated from immunized rabbits. ETX specificity and sensitivity were evaluated via western blot, ELISA, immunocytochemistry (ICC), and flow cytometry. ETX-neutralizing function was evaluated both in vitro and in vivo. All antibodies recognized both purified ETX and epsilon protoxin via western blot with two capable of detecting the ETX-oligomer complex. Four antibodies detected ETX via ELISA and three detected ETX bound to cells via ICC or flow cytometry. Several antibodies prevented ETX-induced cell death by either preventing ETX binding or by blocking ETX oligomerization. Antibodies that blocked ETX oligomerization inhibited ETX endocytosis and cellular vacuolation. Importantly, one of the oligomerization-blocking antibodies was able to protect against ETX-induced death post-ETX exposure in vitro and in vivo. Here we describe the production of a panel of rabbit monoclonal anti-ETX antibodies and their use in various biological assays. Antibodies possessing differential specificity to ETX in particular conformations will aid in the mechanistic studies of ETX cytotoxicity, while those with ETX-neutralizing function may be useful in preventing ETX-mediated mortality.

## 1. Introduction

Epsilon toxin (ETX), produced by *Clostridium perfringens* (*C. perfringens*) toxinotypes B and D, is responsible for causing enterotoxaemia, an economically devastating disease in sheep and other ruminant livestock [1,2]. Currently, ETX is the third most lethal bacterial toxin with an estimated LD50 of 100 ng/kg in mice [3]. As a result, ETX is listed as a category B bioterrorism agent by the Center for Disease Control (CDC). ETX is toxic to specific human cell lines [4,5,6,7] and is suggested to be a possible environmental trigger for multiple sclerosis (MS) in humans [8,9,10].

ETX is secreted by *C. perfringens* type B and D during exponential growth as a relatively weak, 33 kDa protoxin (pETX). Enzymatic activation by the proteases trypsin, chymotrypsin, and lambda toxin increases its potency one thousand-fold. Each enzyme cleaves at distinct amino acid residues at both the C and N termini, producing active toxin approximately 27 kDa in size. Interestingly, maximum potency is achieved when pETX is activated with both trypsin and chymotrypsin [11,12,13]. Importantly, cleavage at the C-terminus is essential for toxicity [11].

ETX is a member of the aerolysin-like pore-forming toxin family, with cytotoxicity thought to be a result of heptameric pore formation. ETX pore formation is believed to occur in three stages: (1) binding of ETX to its cell surface receptor, (2) ETX oligomerization on the cell surface (pre-pore complex), and (3) insertion of the ETX-oligomer into the plasma membrane, creating a functional pore [14]. The myelin and lymphocyte protein MAL appears to be the most likely ETX receptor [7,15], although other receptors including the Hepatitis A Virus Cellular Receptor 1 (HAVCR1) [6] have been suggested. In addition, caveolin-1 and caveolin-2 are important for ETX oligomerization, but not binding [16]. Formation of a functional pore results in rapid cell death via membrane permeability, ATP depletion, and mitochondrial dysfunction [16,17,18,19,20,21]. Pore formation results in a rapid influx of K and rapid efflux of Cl^−^ and Na^+^, followed by a slower increase in intracellular Ca^2+^ [19]. The pore is slightly anionic [19] and asymmetrical in shape [22]. At the cell surface, the extracellular side of the pore is estimated to be 0.4 nm in diameter, allowing passage of 500 Da molecules. On the cytoplasmic side, the diameter is believed to be 1.0 nM, allowing passage of molecules as large as 2300 Da.

Active ETX is comprised of three domains, each with a critical role in ETX binding and cytotoxicity. Domain I contains numerous aromatic amino acids and the sole tryptophan residue, which contributes to receptor binding [3,23]. Single point mutations within this domain inhibit binding to susceptible cells [24,25,26,27,28,29,30,31,32,33]. Domain II is believed to play an important role in toxin oligomerization, stabilization, and insertion into the membrane [23,31,32]. Mutations within this domain reduce or inhibit cytotoxicity without affecting ETX binding. Domain III, which contains the C-terminus, is also important in membrane insertion and oligomerization as mutations in domain III block ETX oligomerization [23,30]. As suggested by previous experiments [34,35], it is plausible that antibodies directed against external epitopes in any of ETX’s three domains could neutralize cytotoxicity either by blocking ETX binding or oligomerization and pore formation.

To investigate if ETX may be an environmental cause of MS in humans, we sought to generate highly sensitive monoclonal anti-ETX antibodies capable of detecting low levels of ETX in various biological samples using diverse techniques. Although other anti-ETX antibodies have been generated and used for both detection and neutralization [35,36,37,38,39,40,41,42], we required large amounts of these antibodies to perform a clinical trial looking for ETX in MS patients versus healthy controls in a multitude of assays. Therefore, it made more economical and logistical sense to produce these antibodies ourselves. In addition, we also sought to produce rabbit monoclonal antibodies because rabbit monoclonals are believed to have higher antigen affinity and more robust results in various assays compared to mouse monocolonals [43,44,45,46]. In addition, monoclonal antibodies have less background complication compared to anti-sera or even affinity-purified polyclonal antibodies [47,48,49].

In this paper, we describe generation of seven anti-ETX rabbit monoclonal antibodies and identify which of these antibodies are suitable for various immunoassays including: western blot, immunocytochemistry (ICC), and flow cytometry for detection of ETX and pETX on the ETX-susceptible CHO cell line expressing a rat MAL fusion protein (rMAL-CHO) [15]. The suitability of these rabbit monoclonals for different applications is summarized in Table 1. Importantly, we also identify monoclonal antibodies capable of neutralizing ETX cytotoxicity by blocking ETX binding or oligomerization both in vitro and in vivo. Excitingly, we present a toolbox of seven anti-ETX monoclonal antibodies that may have the potential to (1) confirm the presence of ETX in various biological assays, (2) aid in the mechanistic research of ETX-induced cytotoxicity, and (3) neutralize ETX cytotoxicity.

## 2. Materials and Methods

### 2.1. Epsilon Protoxin

pETX was provided by BEI (Biodefense and Emerging Infections Research Resources Repository, Manassas, VA, USA) at a minimum >95% purity at 0.5 mg/mL (Epsilon Protoxin, from *Clostridium perfringens*, Strain 34 Type B, NR-856). pETX was labeled with Alexa Fluor 647 and Protein Labeling Kit (Life Technologies, Carlsbad, CA, USA) as per manufacturer’s instructions (pETX-647). Labeled toxin was stored in a 50% glycerol stock (10 μM) at −80 °C until use. Alternatively, pETX was labeled with Alexa Fluor 594 and Protein Labeling Kit (Life Technologies) as per manufacturer’s instructions, aliquoted and stored at −80 °C until use.

### 2.2. Activation of ETX

Unlabeled pETX from BEI was activated in house using immobilized trypsin, TPCK Treated, agarose resin (Thermo Fischer Scientific, Waltham, MA, USA). Briefly, 125 μL resin was washed three times in sodium phosphatase buffer (pH 7.98). Resin was suspended in 200 μL sodium phosphatase buffer and combined with 500 μL of BEI proETX (0.5 mg/mL) for two hours at 37 °C with gentle agitation. The solution was centrifuged at 18,000 rcf for 10 min and the supernatant containing the activated ETX collected. Activated toxin (11 μM) was aliquoted and stored at −80 °C until use. Alexa Fluor 594 labeled proETX was activated similarly (ETX-594). Fluorescent labeling did not alter ETX cytotoxicity or binding.

### 2.3. Rabbit Immunization

An adult, New Zealand White, female rabbit was used for immunization. Pre-immune sera were collected prior to immunization. The animal was immunized three times subcutaneously with pETX with TiterMax Classic Adjuvant (Sigma, St. Louis, MO, USA). For the first immunization, the animal received 0.025 mg/kg pETX. One month later, the animal received a second immunization of 0.1 mg/kg. Sero-reactivty to ETX was determined two weeks after the second immunization. Five weeks after the second immunization, the animal received its third immunization of 0.008 mg/kg pETX. Two weeks after the third immunization, 30 mL of blood was harvested from the animal for peripheral blood mononuclear cells (PBMC) isolation.

### 2.4. Affinity Purification of Rabbit Polyclonal Antibodies against pETX

Polyclonal antibodies were purified from immunized rabbit sera (two weeks after second immunization) using AminoLink Plus Immobilization Kit (Thermo Fischer Scientific) per the manufacturer’s instructions. pETX was immobilized to agarose beads to isolate anti-ETX antibodies from serum.

### 2.5. Rabbit PBMC Isolation

Blood samples were collected from immunized rabbit in Becton Dickinson Vacutainer K2 EDTA 7.2 mg blood collection tubes. Samples reached room temperature and were then diluted with an equal volume of Phosphate Buffered Saline (PBS) and 2% Fetal Bovine Serum (FBS). Diluted blood was layered on top of Ficoll-Paque PLUS (GE Healthcare Biosciences, Marlborough, MA, USA). Tubes were centrifuged at 1200 rcf for 20 min at room temperature (without brakes). Buffy coat containing PBMC was collected using a sterile transfer pipet. PBMC were stored at −80 °C until use.

### 2.6. B-Cell Panning

B-cells expressing anti-ETX antibodies were isolated using immuno-panning. A 96-well flat bottom cell culture plate was coated with 2 µg/mL pETX in 50 µL PBS overnight (ON) at 4 °C. Wells were washed with 200 µL PBS and incubated in 200 µL base media (RPMI 1640 with 10% heat-inactivated FCS, 2 mM l-glutamine, 10 mM HEPES, 100 units/mL penicillin, 100 µg/mL streptomycin and 55 µM 2-mercaptoethanol) for 1 h at room temperature to block non-specific sites. Media was aspirated prior to addition of PBMC. PBMC were added to wells at concentrations ranging 2500 and 5000 cells/well in base media and incubated for 2 h at 37 °C in 5% CO_2_ in a humidified incubator. During the development of the protocol, the number of PBMC added to each well was titrated to find the cell number range that would favor a clonal B-cell population and ensure that the vH and vL sequences retrieved, belong to a single specific antibody. Recovery of multiple vH and vL with unique sequences from the same well is rare, but the multiple sequences can still be paired and expressed to determine which vH/vL pairing produces the correct antibody. Plates were washed three times with base media to remove any non-adhered cells. The plate bound B-cells were cultured for 7 days with conditions that promote differentiation and proliferation as previously described in Lightwood et al. [50]. Plates were incubated for seven days at 37 °C in 5% CO_2_ in a humidified incubator. Supernatants were screened for anti-ETX activity using indirect ELISA and flow cytometry (described below). B-cells from anti-ETX positive wells were removed by thorough washing with PBS containing RNase Inhibitor (Roche, Basel, Switzerland). Cells were centrifuged at 290 *g* for 10 min with a swinging bucket rotor. Supernatant was aspirated, and cell pellets frozen at −80 °C until further use.

### 2.7. B-Cell Cloning and Monoclonal Antibody Production

Frozen B-cells were thawed on ice for 10–15 min. RNA extraction was performed using the QIAshredder columns and Qiagen RNeasy Plus Micro kit (Qiagen, Hilden, Germany) per the manufacturer’s protocol. cDNA synthesis was performed using Invitrogen SuperScript III First strand synthesis supermix for qPCR according to manufacturer’s protocol. To polymerase chain reaction PCR amplify the vH and vKappa gene sequences, a proprietary set of primers (Biogen, Cambridge, MA, USA) was used. PCR was performed using Invitrogen Platinum Taq Hi-Fi polymerase (Invitrogen, Carlsbad, CA, USA). The PCR reaction steps were as follows: 94 °C for 1 min, 94 °C for 15 s, 56 °C for 30 s, 68 °C for 50 s, repeat from step two 30 times, and 68 °C for 8 min. PCR amplification was confirmed by gel electrophoresis. Successful PCR reactions were cloned into pCR4 via TOPO/TA cloning using Invitrogen TOPO TA Cloning Kit for Sequencing kit (Invitrogen, Carlsbad, CA, USA) per the manufacturer’s protocol. Cloned products were transformed into NEB5a chemical competent *E. coli* cells (New England Biolabs, Ipswich, MA, USA) per the manufacturer’s instructions. Transformed *E. coli* cells were plated onto Luria-Bertani LB agar plates containing 100 μg/mL ampicillin. Resistant colonies from the heavy and light chain clones were inoculated into 1.2 mL of Terrific broth in 96 well deep well dishes. Cultures were grown at 37 °C for 24 h with shaking at 750 rpm. Plasmid DNA was isolated using Qiagens miniprep using the QIAGEN robot kit (Qiagen, Catalog#962141) on QIAGEN Universal robot. DNA was transferred to 96-well plates and submitted for sequencing via Applied Biosystems 3730xl DNA Analyzer (Applied Biosytems, Foster City, CA, USA). Appropriate sequences were sent to ATUM (Formerly DNA2.0, formerly MIGS, LCC; Newark, CA, USA) and cloned into a MIGS expression vector (propriety vector) for antibody production in human embryonic kidney (HEK) cells. Antibodies were purified by KanCap A resin. All antibodies are IgG1 and were supplied to our lab at known concentrations in PBS.

### 2.8. rMAL-CHO Cell Culture

CHO cells stably expressing rMAL-GFP fusion proteins were generated as previously described [15]. rMAL-CHO cells were maintained and treated with indicated doses of ETX in Dulbecco’s Modified Eagle’s Medium/Ham’s F12 medium (Life Technologies) with 10% heat-inactivated FBS, 50 units/mL penicillin and 50 μg/mL streptomycin and supplemented with 1X GlutaMAX (Thermo Fisher Scientific).

### 2.9. rMAL-CHO Cell Lysates from Western Blot Analysis

rMAL-CHO cells grown to 80–90% confluence in 6 or 12 well dishes and treated with indicated doses of ETX in 2 or 1 mL of media, respectively, for the indicated time at 37 °C in 5% CO_2_ in a humidified incubator. Cells were moved to ice, then washed three times with ice cold PBS. Cells were lysed in ice-cold RIPA buffer (50 mM Tris-HCl (pH 8.0), 150 mM NaCl, +1% NP-40, 0.1% Sodium dodecyl sulfate (SDS), 0.5% Sodium Deoxycholate) with proteinase and phosphatase inhibitors (Cell Signaling Technologies, Danvers, MA, USA) for 10 min. For 6 well plates, cells were lysed in 1 mL of RIPA buffer, for 12 well plates, they were lysed in 0.5 mL. Samples were centrifuged at 5000 rcf for 5 min to pellet nuclei and DNA. Supernatants were collected and used for western blot analysis. In select experiments, media containing 25 nM ETX were pre-incubated with anti-ETX antibodies at 15 µg/mL for 30 min at 37 °C in microcentrifuge tubes prior to rMAL-CHO cell treatment.

### 2.10. Western Blot (WB) Analysis and Densitometry Measurements

5 ng of pETX and active ETX in PBS were used as positive controls. Whole-cell lysates from ETX-treated rMAL-CHO cells were used to detect ETX-pore and whole-cell lysates from untreated rMAL-CHO cells were used as negative controls. All samples were prepared in 2X Laemmli Sample Buffer (Bio-Rad, Hercules, CA, USA) containing 5% 2-Mercaptoethanol (Bio-Rad) and heated at 95 °C for 5 min before loading onto 4–20% Mini-PROTEAN TGX Stain-Free gels (Bio-Rad). Gels were run in Tris/Glycine SDS Buffer (Bio-Rad) at 200 V for 35 min. Semi-dry transfers were performed in transfer Tris/Glycine Buffer (Bio-Rad), using the Trans-Blot SD Semi-Dry Electrophoretic Transfer Cell system (Bio-Rad) at 15 V for 15 min. Blots were blocked in 5% Blotting-Grade Blocker nonfat milk (Bio-Rad) in Tris Buffered Saline with Tween 20 (TBS-T, Cell Signaling Technology) for one hour at room temperature. Blots were then incubated with primary antibodies JL001.1, JL001.2, JL002, JL004, JL005, JL006, or JL008 at 0.34 µg/mL in blocking solution overnight at 4 °C. Blots were washed 4 times for 5 min in TBS-T at room temperature, and incubated with secondary antibody peroxidase-conjugated Affinipure Goat Anti-Rabbit IgG H + L (Jackson ImmunoResearch, West Grove, PA, USA) at 0.024 µg/mL in blocking solution for 2 h at room temperature. Blots were washed 4 times for 5 min in TBS-T and developed for 5 min at room temperature in SuperSignal West Dura Extended Duration Substrate (ThermoFisher Scientific). The developed blots were visualized on 5 × 7 CL-XPosure Films (ThermoFisher Scientific) at various exposure times using a Konica Minolta SRX-101A film processor. Densitometry measurements were taken from scanned films using ImageJ64 software (National Institutes of Health, Bethesda, MD, USA) and measurements were analyzed in Prism 7 software (GraphPad, San Diego, CA, USA).

### 2.11. Indirect ELISA

Thermo Scientific Nunc MaxiSorp TM flat-bottom ELISA plates were incubated ON at 4 °C with 50 µL of the following amounts of active ETX: 0.3125 nM, 0.625 nM, 1.25 nM, 2.5 nM, 5 nM, 10 nM, 20 nM in 0.1 M coating buffer (Fisher Scientific Sodium Bicarbonate Crystalline Powder solubilized in DI H20). Plates were washed three times with wash buffer (0.1% Tween in PBS) and blocked with 150 µL of blocking buffer (Thermo Scientific Blocker Casein diluted in wash buffer) for 1 h. 50 µL of antibodies at 1 µg/mL in blocking buffer were incubated in coated and blocked wells for 1 h at RT. Plates were washed three times with wash buffer. Antibody binding was determined using 50 µL of peroxidase-conjugated Affinipure Goat Anti-Rabbit IgG (H + L) (Jackson ImmunoResearch) diluted 1:10,000 in blocking buffer, at RT for 45 min. Plates were washed four times with wash buffer for 30 s. Peroxidase activity was measured with 50 µL of BioFX TMB (Surmodics, Eden Prairie, MN, USA) solution for 10 min. Immediately after, reaction was stopped with 50 µL of 2N sulfuric acid and absorbance at 450 nm read using BioTek’s Synergy HTX multi-mode reader and Imager Software (BioTek, Winooski, VT, USA). Absorbance readings were analyzed in Microsoft Excel. In select experiments, plates were coated with 1, 10, 100, and 1000 nM of pETX and probed with 1 µg/mL of anti-ETX antibodies. In other experiments, plates were coated with 10 nM pETX and probed with 0.1, 1, 10, and 100 µg/mL of anti-ETX antibodies. The same buffers and incubation times were used as preciously described. For select experiments, the limit of detection was determined by taking the blank (0 nM) mean and adding the standard deviation multipled by three. The limit of detection (LOD) was determined by taking the blank (0 nM) mean plus three times the standard deviation (Mean Blank ± 3*STDEV).

### 2.12. Sandwich ELISA

96-well Nunc-immuno Maxisorp plates (Sigma) were coated with 50 µL of 1 µg/mL of JL004 rabbit capture antibody overnight at 4 °C in coating buffer (0.1 M sodium bicarbonate in water). Wells were blocked for 1 h at room temperature using casein solution (Thermo Fischer Scientific) diluted 1:10 in wash buffer (PBS containing 0.1% Tween 20). pETX capture entailed 50 µL of solution containing analyte incubated for 2 h at room temperature. 50 µL of detection antibody (2 µg/mL of JL001.2 rabbit with mouse Fc region) was incubated for 1 h at room temperature. For secondary antibody binding, 50 µL of secondary antibody (horse radish peroxidase-conjugated goat anti-mouse, Jackson ImmunoResearch) diluted 1:10,000 in blocking buffer was added to wells for 45 min. Wash steps using wash buffer were performed after each of these steps. ELISA development involved addition of 50 µL of Bio FX TMB (Surmodics) substrate solution for 10 min in the dark followed by addition of 50 µL of 2N sulfuric acid. BioTek’s Synergy HTX multi-mode reader and Imager Software. Absorbance at 450 nm read using BioTek’s Synergy HTX multi-mode reader and Imager Software. Absorbance readings were analyzed in Microsoft Excel and Prism 7 software. The LOD was determined by taking the blank (0 nM) mean plus three times the standard deviation (Mean Blank ± 3*STDEV).

### 2.13. Immunocytochemistry (ICC)

rMAL-CHO cells were seeded onto Poly-d-Lysine/Laminin coated Corning BioCoat 12 mm coverslips (Thermo Fisher Scientific) and allowed to grow overnight in a 24 well dish. Cells were treated with or without 50 nM ETX for 30, 60, or 120 min. Cells were washed in PBS then fixed in 4% PFA for ten minutes. Cells were blocked in 10% FBS in PBS with 0.1% triton-100 (Sigma) for 30 min. Cells were stained overnight at 4 °C with 5 ug/mL of antibody in the same buffer. In select experiments, cells were stained for Rab7 (Colne D95F2, XP^®^ Rabbit mAb #9367, Cell Signaling Technology) at a 1:200 dilution overnight. Cells were washed in PBS and bound antibody detected using Cy3 conjugate goat anti-rabbit (Jackson ImmunoResearch) diluted 1:500 in 5% FBS in PBS with 0.05% triton-100 for 1 h at room temeprature. Cells were washed with PBS and then mounted onto microscope slides with VECTASHIELD Antifade Mounting Medium with DAPI (Vectorlabs, Burlingame, CA, USA). Cells were imaged using Zeiss Axioskop2 Plus upright microscope (Oberkochen, Germany) and Spot RT3 camera and software (Spot Imaging, Sterling Heights, MI, USA). Images were post-processed using Adobe Photoshop (Adobe, San Jose, CA, USA). For relative fluorescence measurements, images were imported into ImageJ64 and converted into an 8-bit gray format. The threshold was identically adjusted for all anti-ETX images and converted to a binary image. The mean and integrated density was calculated for each individual image and was used as a measurement of fluorescence density. In addition, the number of nuclei per field were analyzed using the Analyze Particles function. Fluorescent density and nuclei count were exported to Microsoft Excel and the relative fluorescent density calculated by dividing the fluorescent density by the number of nuclei per field. The relative fluorescent densities for each antibody were analyzed in Prism 7 software. Alternatively, rMAL-CHO cells seeded on coverslips were also treated with fluorescently labeled ETX-594. In these experiments, media containing 50 nM ETX-594 were pretreated with 15 μg/mL of antibodies for 30 min at 37 °C for 30 min prior to cell treatment. For these experiments, cells were fixed in 4% PFA for 10 min, washed with PBS, and directly mounted onto microscope slides.

### 2.14. Flow Cytometry

rMAL-CHO cells were grown in 6-well dishes to 90–100% confluence. Cells were treated with or without 50 nM ETX in 2 mL of media for 1 h. Cells were then washed and trypsinized to suspend cells. Trypsinized cells were fixed in 2% PFA for ten min, then blocked in 10% FBS in PBS for 30 min. Control and ETX-treated cells were both stained with individual antibodies in cell staining buffer at 0.2 μg/mL (Biolegend, San Diego, CA, USA). Cells were also stained with a monoclonal isotype control (Abcam, Clone EPR25A, Cambridge, UK) at 0.2 μg/mL. Finally, cells were also stained with AP204 at 1:100 dilution and Normal Rabbit Serum Control (Invitrogen) was used neat as a polyclonal isotype control. Cells were stained for 30 min at RT. Cells were washed in PBS + 0.2%FBS, and antibody binding determined using a phycoerythrin PE conjugated anti-rabbit antibody (Thermo Fisher Scientific). Cells were incubated with PE conjugate Donkey anti-rabbit IgG (minimal x-reactivity) antibody (Biolegend) at 0.4 μg/mL for 30 min at RT. Cells were washed in PBS and analyzed on a FACSVerse (BD Biosciences, San Jose, CA, USA) and analyzed using FlowJo software (FlowJo, LCC, Ashland, Oregon, USA). Alternatively, media containing 50 nM of pETX-647 was pre-incubated with 15 μg/mL of anti-ETX antibodies for 30 min at 37 °C prior to treatment of rMAL-CHO cells.

### 2.15. Cell Death Assays with rMAL-CHO Cells

rMAL-CHO cells were seeded into 96 well cell culture treated dishes. In some experiments, media with and without 100 nM ETX was pre-incubated with indicated amounts of anti-ETX antibodies at 37 °C for 30 min. Pretreated media was used to treat rMAL-CHO cells for 1 h or 2 h as indicated. Cell death was then analyzed by propidium iodide (PI) inclusion assay. Cells were treated with 50 μg/mL of PI and live images of randomly chosen fields in each well were acquired under an inverted fluorescence microscope (Nikon, Minato, Tokyo, Japan) equipped with a Charged Coupled Device (CCD) camera (Carl Zeiss, Oberkochen, Germany) and were then imported into ImageJ64 in 8-bit gray format. For quantification of PI-positive cells, the images were converted into binary images by applying the same threshold value to all images collected from the same experiment. Analyze Particles function was selected to automatically count the particle numbers and to analyze particle properties, such as size shape, and distribution patterns. Data were exported and analyzed in Excel. In other experiments, rMAL-CHO cells were treated with 100 nM of ETX and anti-ETX antibodies JL004 and JL008 were spiked in at specific time points post-ETX treatment. Cell death was measured using the same method described above.

### 2.16. In Vivo Mouse Experiments

All animal work was conducted according to federal guidelines and approved by the Weill Cornell Medical College Institutional Animal Care and Use Committee. C57BL/6J mice were intravenously infused with 0.1 mg JL008 in saline either 30 min prior to ETX treatment or one hour post-ETX treatment. Mice intravenously infused with saline were used as controls. Mice were treated with 5 ng per gram of body weight ETX via intraperitoneal injection. Mice were observed for up to 360 min for moribund neurological behavior including seizures, extreme unbalance, and extreme lethargic behavior (not moving when probed). Moribund mice were euthanized using a lethal dose of ketamine/xylazine cocktail.

## 3. Results

### 3.1. Production of Monocolonal Antibodies Against ETX

**Figure 1:**
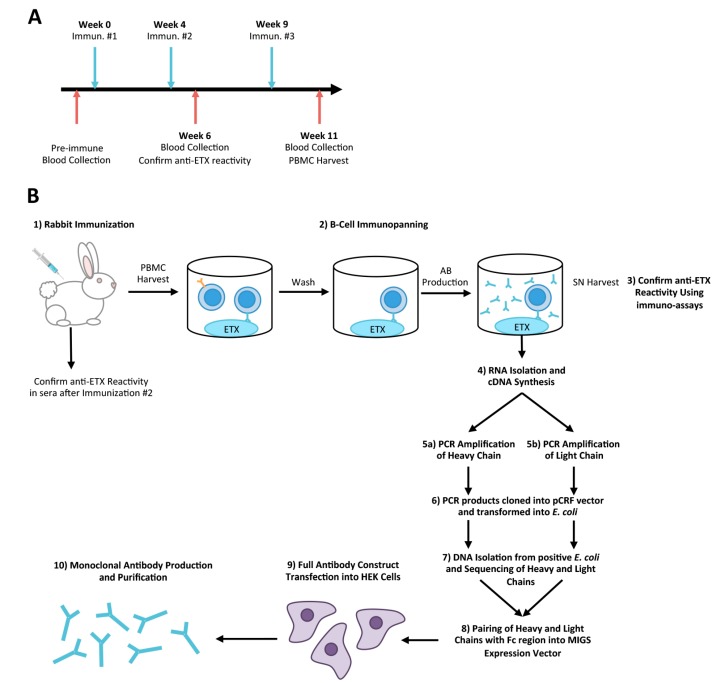
Overview of Rabbit immunization and production of anti-ETX monoclonal antibodies. (**A**) Timeline for rabbit immunization with pETX and blood collection. (**B**) Overview of rabbit B-cell isolation and cloning procedure for production of anti-ETX monoclonal antibodies. For detailed information, please referee to the methods section.

Detailed descriptions of this process can be found in the materials and methods section. Figure 1 briefly outlines the process from immunization to generation of monoclonal antibodies. A White New Zealand rabbit was immunized twice with pETX plus adjuvant one month apart (Figure 1A). Anti-ETX positivity was checked in the rabbit sera and the rabbit was immunized a third and final time five weeks after the second immunization. Two weeks later, 30 mL of blood was harvested and PBMC isolated. To select for pETX-specific B-cells, immuno-panning against immobilized pETX was performed in cell culture plates (Figure 1B) [50]. B-cells were cultured for one week and harvested supernatant was evaluated for ETX reactivity using flow cytometry and indirect ELISA. cDNA was synthesized from positive wells and subsequently cloned to produce full-length antibodies. In total, 120 immunopanned B-cell wells were tested. Of these 120 wells, 10 positive wells were selected for cloning. From these 10 positive wells, seven rabbit IgG1 monoclonal antibodies were produced: JL001.1, JL001.2, JL002, JL004, JL005, JL006, and JL008. Polyclonal rabbit antibodies were also affinity-purified from immunized rabbit serum using immobilized pETX.

### 3.2. Evaluation of Anti-ETX Antibodies for Detection of pETX, ETX, and ETX-Oligomer Complex via WB

**Figure 2:**
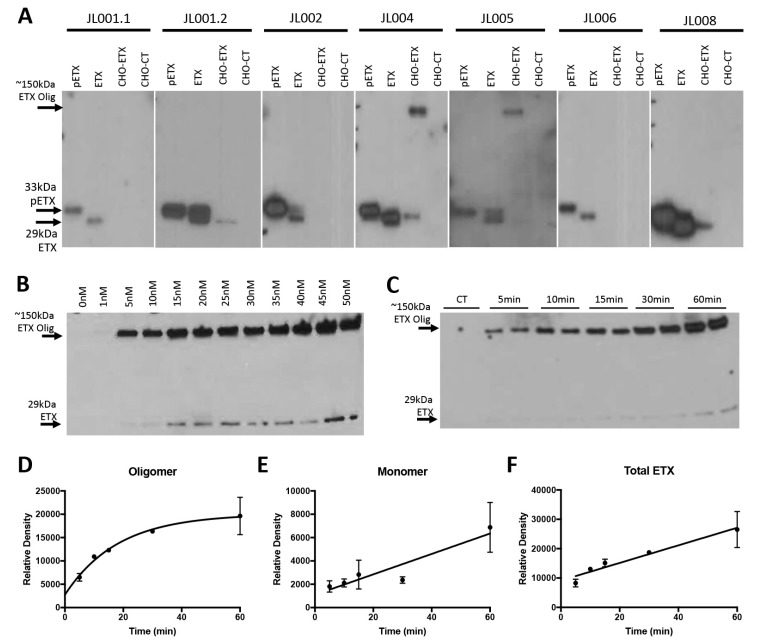
Detection of pETX, ETX, and ETX-oligomer via western blot analysis. (**A**) 5 ng of ETX and pETX were loaded onto gels. To detect ETX-pore, whole-cell lysates from rMAL-CHO cells (CHO-ETX) were used. Whole-cell lysates from untreated rMAL-CHO cells (CHO-CT) were used as a negative control. Antibodies JL001.1 and JL006 only detected purified pETX and ETX. JL1001.2 and JL008 were able to detect pETX and ETX, as well as bound ETX monomer of treated rMAL-CHO cells. JL005 was able to detect pETX and ETX as well as the ETX-oligomer. JL004 was able to pETX, ETX, ETX monomer bound to rMAL-CHO cells, and the ETX-oligomer. JL004 was used to determine a dose response (**B**) and time course (**C**) for ETX pore formation. For the dose response curve, cells were treated for 30 min at indicated ETX doses. For the time course, cells were incubated with 50 nM of ETX for indicated time points. 0 min time point is untreated control. Results for time course are shown in duplicate. (**D**) Densitometry measurements for ETX pore formation over time. (**E**) Densitometry measurements for bound ETX monomer over time. (**F**) Densitometry measurements for total ETX (Pore + Monomer) detected over time. Results are mean ± SD.

To determine the sensitivity and specificity of anti-ETX monoclonal antibodies, we tested reactivity against ETX (29 kDA) and pETX (33 kDa) by WB analysis (Figure 2A). Detection of the ETX-oligomer complex, sometimes referred to as the ETX-pore complex, was also evaluated. To detect the ETX-oligomer, whole-cell lysates from ETX-treated rMAL-CHO cells were used [15]. This cell line is known to be ETX-sensitive and to form ~150 kDA ETX-oligomer complex. Whole-cell lysates from untreated rMAL-CHO cells were used as negative controls. All membranes were probed with 0.34 μg/mL of each antibody. JL001, JL002, and JL006 successfully detected purified ETX and pETX at their correct molecular weights. JL004 and JL005 were able to detect purified ETX and pETX as well as the ETX-pore complex. Interestingly, JL004 also detected a band at 29 kDA in the ETX-treated rMAL-CHO cells. As ETX-treated rMAL-CHO cells were thoroughly washed before lysis, this band is believed to be bound ETX monomer on treated rMAL-CHO cells. Although JL005 produced a high amount of background, a faint band corresponding to the bound ETX monomer can also be detected. Surprisingly, JL001.2 and JL008 also detected bound ETX monomer in rMAL-CHO cells, but did not recognize the ETX-oligomer. JL001.2 and JL008 also both detected purified ETX and pETX.

To determine the extent of JL004 sensitivity for ETX-oligomer detection; a dose response curve was evaluated (Figure 2B). rMAL-CHO cells were incubated with 0, 1, 5, 10, 15, 20, 25, 30, 35, 40, 45, and 50 nM ETX for 30 min prior to lysing. Strong ETX-oligomer detection was achieved at 5 nM ETX treatment and increased with ETX dose. A weak signal for the ETX-oligomer was detected at 1 nM ETX treatment, which became more obvious with longer film exposure (data not shown). Interestingly, a strong signal for bound ETX monomer was detected at 15 nM. This signal increased proportionally with ETX dose. Weak bound ETX monomer signals were detected at 5 nM and 10 nM ETX doses as well. The observation that ETX oligomerization is observed after 1 and 5 nM ETX treatment indicates that only a relatively small amount of ETX is needed to cause ETX oligomerization in this cell line.

JL004 was also used to study ETX oligomerization over time when cells were treated with 50 nM ETX via WB. ETX oligomerization was rapid, occurring 5 min after ETX exposure and increasing with time (Figure 2C). Weak detection of bound ETX monomer was achieved at 5 min and increased with time. Densitometry reading revealed that ETX oligomerization followed an inverse exponential curve (Figure 2D) while bound ETX monomer followed a linear curve (Figure 2E) with *r*^2^ values of 0.9136 and 0.8624, respectively. However, when total ETX incorporation into rMAL-CHO cells was evaluated by adding the densitometry readings for ETX-oligomer plus the densitometry readings for bound ETX monomer, the relationship appeared linear with an *r*^2^ value of 0.9504. When examined for a non-linear relationship, the *r*^2^ was 0.8797. Taken together, this data suggests that total ETX binding may follow a linear process but ETX monomer is rapidly and exponentially oligomerized. It appears that ETX oligomerization can be saturated. This may be due to limited receptor or co-receptor components needed for ETX pore formation.

### 3.3. Evaluation of Anti-ETX Antibodies for Detection of ETX in Indirect ELISA

**Figure 3:**
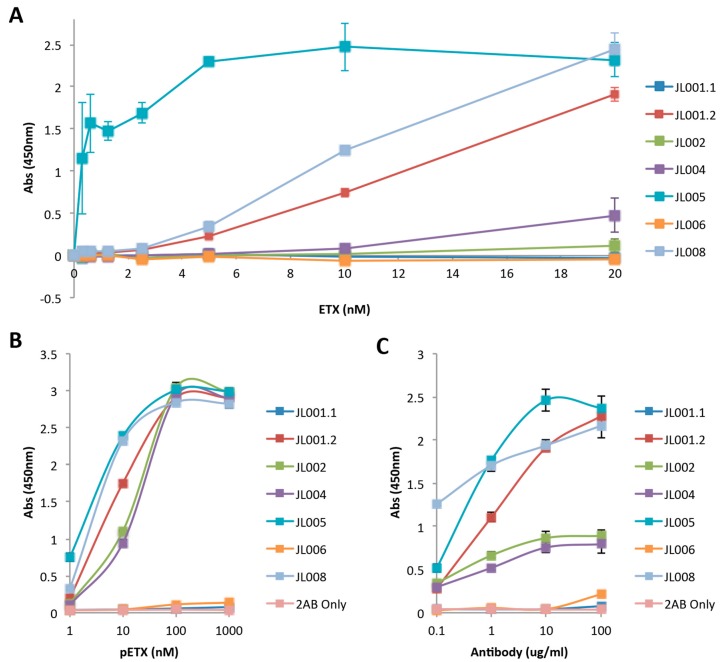
Detection of ETX via indirect ELISA. (**A**) ELISA plates were incubated with indicated amounts of ETX and then probed with 1 μg/mL of indicated anti-ETX monoclonal antibody. Displayed results are adjusted for background absorbance. (**B**) ELISA plates were coated with indicated amounts of pETX and probed with 1 μg/mL of the specified anti-ETX monoclonal. (**C**) Alternatively, ELISA plates were coated with 10 nM pETX and probed with the specified concentrations of anti-ETX antibodies. Wells probed with peroxidase-conjugated anti-rabbit antibody only (2AB Only) were used as negative controls. Results are mean ± SD.

Sensitivity of monoclonal anti-ETX antibodies was first determined by indirect ELISA (Figure 3A). ELISA plates were incubated overnight with 0.31, 0.63, 1.25, 2.5, 5, 10 and 20 nM ETX. 1 μg/mL of each antibody was incubated with ETX-coated plates for 1 h and binding determined using an HRP-conjugated anti-rabbit antibody. Peroxidase activity was measured using TMB and absorbance measured at 405 nm. JL001.2, JL004, and JL008 successfully detected ETX at these given concentrations within a linear range. Alternatively, JL002 weakly detected ETX. JL001.1 and JL006 were unable to detect ETX at any of the concentrations tested. JL005 produced the strongest signal; however, detection was not within a linear range. In addition, the large variation between replicates observed at the lower ETX concentrations of 0.31 nM and 0.63 nM were considered unfavorable. Experiments were repeated with JL005 at lower antibody concentrations, but high background signal remained an issue (data not shown). This is consistent with the higher amount of background seen in the WBs (Figure 2A). As a result, antibodies JL001.2, JL004, and JL008 were elected the most desirable antibodies for ETX detection via indirect ELISA under these conditions.

To further characterize our antibodies in the indirect ELISA format, experiments were repeated using escalating amounts of pETX with a constant antibody concentration (Figure 3B) or escalating amounts of antibodies with a constant pETX concentration (Figure 3C). Interestingly, JL002 was able to detect pETX levels at higher pETX concentrations while JL001.1 and JL006 failed to detect pETX even at the highest pETX concentrations (Figure 3B). In addition, increasing the antibody concentration for JL001.1 and JL006 failed to detect pETX as well (Figure 3C). Interestingly, increasing the antibody concentration had the biggest effect on pETX detection for JL001.2 and JL005.

### 3.4. Evaluation of Anti-ETX Antibodies for Detection of pETX in Sandwich ELISA

**Figure 4:**
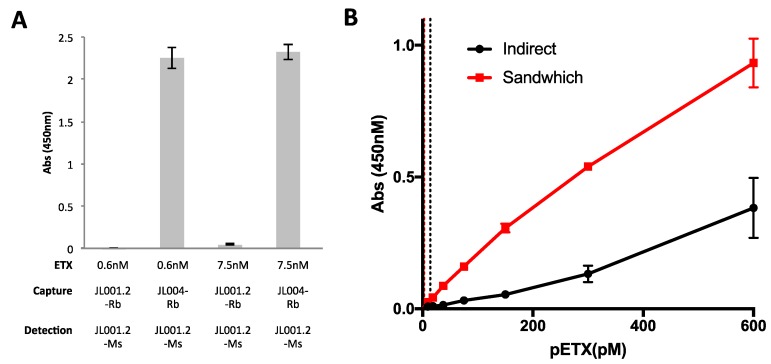
Detection of pETX via Sandwich ELISA. (**A**) To test possible capture antibodies, JL001.2 with the rabbit Fc regions (JL001.2-Rb) and JL004 with the rabbit Fc region (JL004-Rb) were coated on ELISA plates and incubated with 0.6 nM and 7.5 nM pETX. JL001.2 with the mouse Fc region (JL001.2-Ms) was used as the detection antibody. (**B**) Sensitivity of pETX detection using indirect ELISA (JL001.2-Rb) was compared to the sensitivity of the sandwich ELISA using JL004-Rb as capture antibody and JL001.2-Ms as the detection body. The sandwich ELISA was more sensitive than the indirect ELISA method. The limit of detection (LOD) for the sandwich ELISA (red-dotted line) was 2.65 pM and 13.68 pM for the indirect ELISA (black dotted line). Results are mean ± SD.

JL001.2 and JL004 were evaluated for detection of pETX via sandwich ELISA. JL001.2 and JL004 were chosen because they are believed to recognize different epitopes of ETX based on WB analysis (Figure 2A). JL001.2 recognizes purified pETX, ETX, and bound ETX monomer, while JL004 also recognizes the ETX-oligomer. Because JL001.2 and JL004 both contain rabbit Fc regions, the Fab region of JL001.2 was cloned onto a mouse Fc region. For capture of pETX, JL001.2 with rabbit Fc regions (JL001.2-Rb) and JL004 with the rabbit Fc region (JL004-Rb) were used. JL001.2 with the mouse Fc region (JL001.2-Ms) was used as a detection antibody. Both pairs were used to detect 0.6 nM and 7.5 nM of pETX (Figure 4A). As expected, pairing of JL001.2-Rb and JL001.2-Ms failed to detect pETX because of epitope competition. Pairing of JL004-Rb and JL001.2-Ms successfully detected ETX at both 0.6 nM and 7.5 nM pETX.

We then compared indirect versus capture ELISA for the sensitivity of pETX detection using JL001.2-Rb for detection in the indirect format and JL004-Rb as capture antibody with JL001.2-Ms as the detection body in the sandwich format (Figure 4B). The sandwich ELISA was significantly more sensitive than the indirect ELISA method. The LOD for the sandwich ELISA was determined to be 2.65 pM while the LOD for the indirect ELISA was 13.68 pM. The LOD was determined by taking the blank (0 nM) mean and adding the standard deviation multiplied by three (Blank Mean + 3*STDEV). For the sandwich ELISA, the Pearson’s *r* coefficient and *r*^2^ values were 0.9957 and 0.9914, respectively. For the indirect ELISA, the Pearson’s *r* coefficient and *r*^2^ values were 0.9854 and 0.971, respectively.

### 3.5. Evaluation of Anti-ETX Antibodies for Detection of ETX via Immunocytochemistry (ICC)

**Figure 5:**
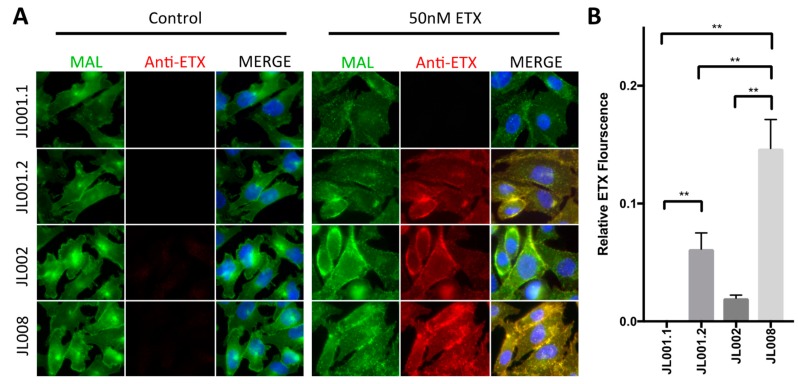
Evaluation of ETX detection via immunocytochemistry (ICC). (**A**) rMAL-CHO cells expressing MAL-GFP fusion protein (green) were treated with or without 50 nM ETX for 30 min. Cells were washed, fixed, blocked, and probed with anti-ETX antibodies. Antibody binding was detected using at Cy-3 conjugated anti-rabbit antibody (red) and visualized by fluorescent microscopy. ETX was detected using JL011.2, J002, and JL008 and colocalized with rMAL-GFP. ETX was not detected with JL001.1 or JL004, JL005, and JL006 (images not shown). (**B**) Relative ETX fluorescence detected on 50 nM ETX-treated rMAL-CHO cells with individual antibodies. JL001.1 was used as a negative control. Results are mean ± SD. ** *p* < 0.01 determined by one-way ANOVA with post-hoc Tukey HSD.

The ability of the rabbit monoclonal antibodies to detect ETX via ICC was evaluated using rMAL-CHO cells (Figure 5). rMAL-CHO cells were treated with 50 nM ETX for 30 min at 37 °C to maximize ETX binding while minimizing ETX internalization. Control and toxin-treated cells were then probed with anti-ETX antibodies and antibody binding determined by Cy3-conjugated anti-rabbit antibody. JL001.2, J002, and JL008 were able to detect ETX via ICC (Figure 5A). ETX treatment of rMAL-CHO cells resulted in internalization of rMAL-GFP, confirming previously published results [15]. In addition, JL001.2, JL002, and JL008 also colocalized with rMAL-GFP, confirming that ETX colocalizes with rMAL-GFP in toxin-treated cells. JL001.1 was unable to detect ETX via ICC. In addition, JL004, JL005, and JL006 were unable to detect ETX via ICC (data not shown). Fluorescence analysis demonstrated that JL008 detected more ETX on ETX-treated cells compared to JL001.2 and JL002 under these conditions (Figure 5B). JL001.1 was used as a negative control. JL001.2 detected significantly more ETX than JL001.1; however, JL002 did not. Taken together, this indicates JL008 is the most suitable antibody for detection of ETX via ICC.

### 3.6. Evaluation of Anti-ETX Antibodies for Detection of ETX via Flow Cytometry

**Figure 6:**
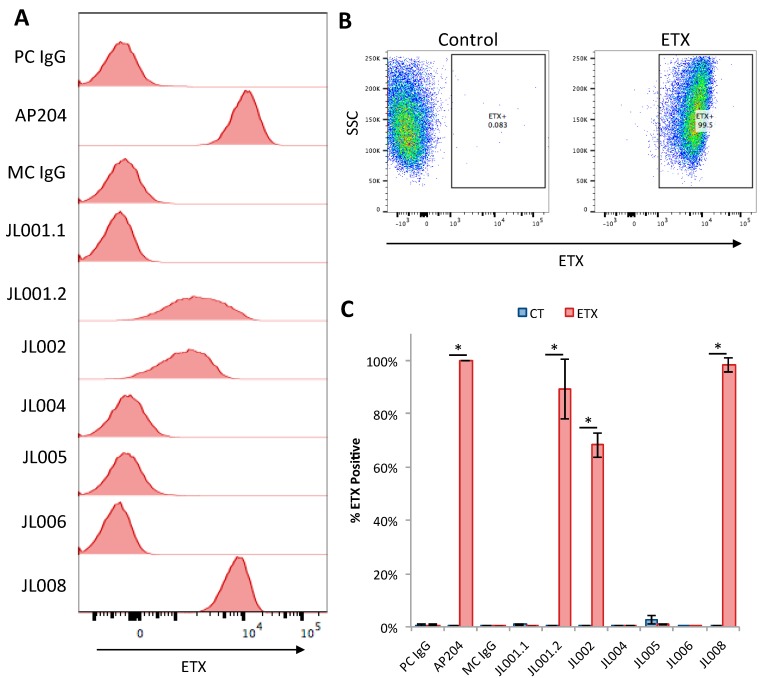
Evaluation of ETX detection via flow cytometry. rMAL-CHO cells were treated with ETX, trypsinized, fixed, blocked, and then probed with anti-ETX antibodies. Bound antibody was detected using a PE conjugated anti-rabbit antibody. (**A**) Histograms of ETX fluorescence for different antibodies. A non-specific polyclonal IgG isotype control (PC IgG) was used a negative control for affinity-purified polyclonal (AP204). A monoclonal IgG isotype control (MC IgG) was used as a negative control for monoclonal antibodies JL001.1, JL001.2, JL002, JL004, JL005, JL006, and JL008. Representative examples of experimental triplicates. (**B**) Representative dot blots of control and ETX-treated rMAL-CHO cells probed with JL008. (**C**) Percent of cells positive for ETX when probed with indicated antibodies. Control treated cells were compared to ETX-treated cells. Results are mean ± SD. * *p* < 0.002 determined by *t*-test.

To determine if monoclonal anti-ETX antibodies would work for ETX detection via flow cytometry, rMAL-CHO cells were treated with 50 nM ETX for one hour. Preliminary experiments demonstrated that this ETX dose and time point achieved the greatest signal (data not shown). Untreated rMAL-CHO cells were used as controls. Cell monolayers were trypsinized to achieve a single cell suspension prior to fixation, blocking, and probing with anti-ETX antibodies. Antibody binding was detecting using a PE conjugate anti-rabbit antibody and evaluated by flow cytometry. CHO cells were selected based on forward scatter (FSC) and side scatter (SSC,) and further gated on rMAL-GFP expressing cells (Supplemental Figure S1). Over 99% of our rMAL-CHO cells expressed rMAL-GFP. A monoclonal isotype control was used as a negative control. Affinity-purified polyclonal antibodies isolated from test bleed number two were also evaluated (AP204). Histogram analysis determined that AP204, JL001.2, JL002, and JL008 all successfully detected ETX on ETX-treated rMAL-CHO cells compared to the isotype control (Figure 6A). AP204 and JL008 had the highest mean fluorescence. ETX fluorescence was also compared to control treated rMAL-CHO cells probed with the isotype control and individual monoclonal anti-ETX antibodies (Supplemental Figure S2). Control treated cells probed with monoclonal anti-ETX antibodies had mean fluorescence similar to the isotype control. AP204 and JL008 had the best separation in fluorescent peaks for all tested controls. Scatter plots for control and ETX treat rMAL-CHO cells probed with JL008 demonstrated that only 0.08% of control cells were within the ETX positive gate, while 99.5% of ETX-treated rMAL-CHO cells were positive for ETX (Figure 6B). When control and ETX-treated cells were probed with individual anti-ETX antibodies, AP204, JL1001.2, JL002, and JL008 identified a significantly higher ETX+ population in the ETX-treated cells compared to control cells (Figure 6C). AP204 detected 100% of ETX-treated cells, JL001.2 detected 89.1%, JL002 detected 68.2%, and JL008 detected 98.2%. Interestingly, JL001.2, JL002, and JL008 were the only monoclonal antibodies to recognize bound ETX via ICC as well. Based on these results JL008 was determined to be the best monoclonal antibody for detection of ETX via flow cytometry.

### 3.7. Neutralizing Ability of Anti-ETX Antibodies

**Figure 7:**
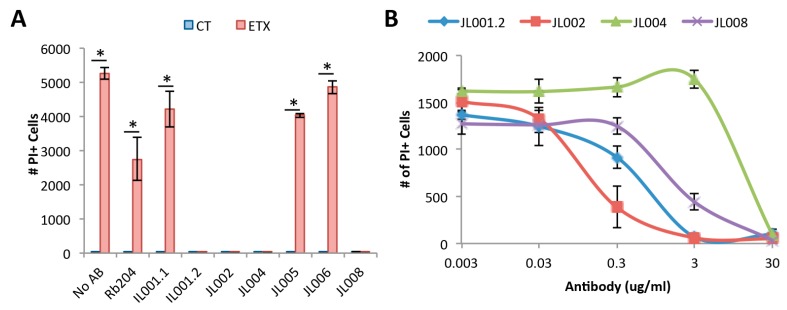
In vitro neutralizing ability of anti-ETX antibodies against ETX. (**A**) Media containing 100 nM of ETX was pre-incubated with the indicated anti-ETX antibodies at 30μg/mL for 30 min prior to treatment of rMAL-CHO cells for 1 h. Media containing antibodies alone were used to evaluate possible antibody toxicity to rMAL-CHO cells (CT). Cells treated without antibodies (no AB) were used as controls. Cell death was evaluated by PI exclusion. Antibodies JL001.2, JL002, JL004, and JL008 prevented cell death similar to non-ETX-treated controls. None of the antibodies exhibited cytotoxic effects on rMAL-CHO cells. * *p* < 0.01 compared to No AB ETX, determined by *t*-test. (**B**) Media containing 100 nM of ETX were pretreated with indicated doses of anti-ETX antibodies before treating cells for 4 h and evaluating cell death by PI exclusion. Results are mean ± SD with a smooth-fit line.

Monoclonal antibodies were evaluated for their ability to inhibit ETX cytotoxicity in rMAL-CHO cells. Media containing 100 nM ETX was pre-incubated with 30 μg/mL of each individual anti-ETX antibody for 30 min prior to rMAL-CHO treatment. 100 nM ETX treatment was chosen to maximize cell death. Media containing antibody alone were used as controls. Cell death was evaluated by promidium iodide (PI) inclusion. rMAL-CHO cells treated with ETX without antibody had significantly higher amounts of PI+ cells compared to untreated control (Figure 7A). A significantly higher amount of cell death was observed in rMAL-CHO cells when ETX containing media was pretreated with AP204, JL001.1, JL005, and JL006 compared to controls, indicating weak or no neutralizing affects. No significant increase in cell death was observed when ETX containing media was pretreated with JL001.2, JL002, JL004, and JL008, indicating that these antibodies neutralize ETX. No significant amount of cell death was observed in any of the controls incubated with antibody alone, indicating that the antibodies themselves have no cytotoxic effects on rMAL-CHO cells.

A titration curve with the four neutralizing antibodies including JL001.2, JL002, JL004, and JL008 was also examined (Figure 7B). Media containing 100 nM ETX were pre-incubated with the indicated antibody concentrations prior to addition to rMAL-CHO cells for four hours. A four-hour time point was chosen to identify maximal neutralizing ability. Interestingly, the four antibodies demonstrated different neutralizing ability. The EC50 of JL001.2, JL002, JL004, and JL008 were 0.36, 0.12, 18.26, and 2.19 μg/mL, respectively.

### 3.8. Anti-ETX Antibodies Neutralize ETX by Blocking ETX Binding and Oligomerization

**Figure 8:**
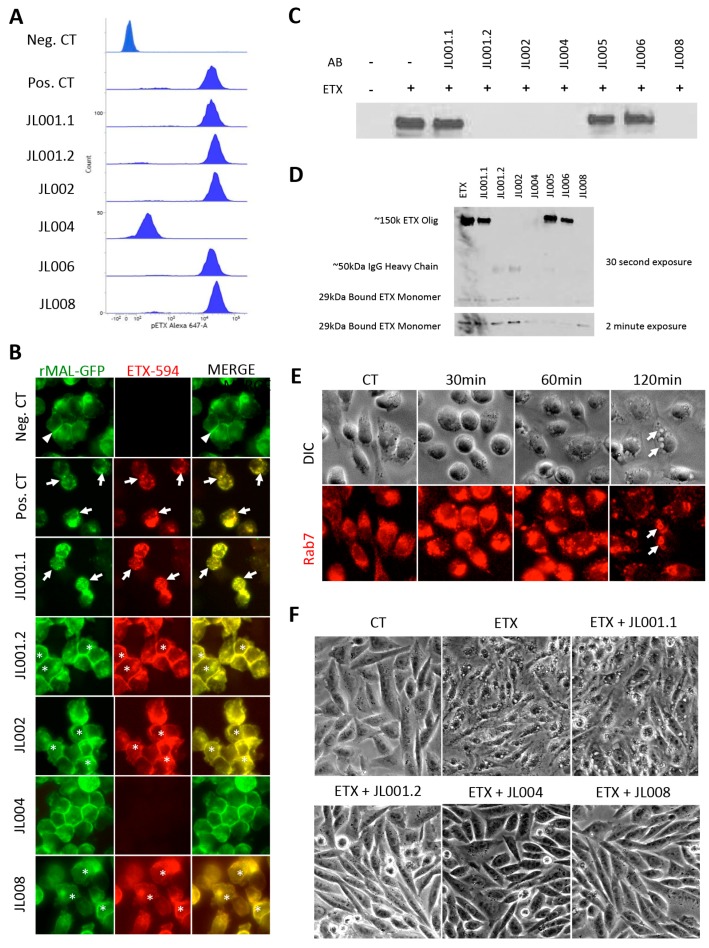
Anti-ETX antibody inhibition of ETX binding and oligomerization in rMAL-CHO cells. (**A**) Alexa-647 conjugated epsilon protoxin (pETX-647) was pre-incubated with indicated anti-ETX antibodies prior to treatment of rMAL-CHO cells. Medium containing pETX-647 alone was used as a positive control for pETX binding (Pos. CT). Medium without pETX or antibodies was used as a negative control (Neg CT). Only antibody JL004 inhibited pETX-647 binding to rMAL-CHO cells. (**B**) Alexa-594 conjugated epsilon toxin (ETX-594) was pre-incubated with indicated anti-ETX antibodies prior to treatment of rMAL-CHO cells. Media containing ETX-594 alone was used as a positive control for ETX binding (Pos. CT). Media without ETX-594 or antibodies was used as a negative control (Neg CT). In the negative control cells, note that the majority of rMAL-GFP is located at the plasma membrane, especially in areas of cell-to-cell contact (white triangles). In comparison, cells treated with ETX-594 alone (Pos. CT) or JL001.1 show evidence of rMAL-GFP internalization, visualized as punctate dots (white arrows). Internalized ETX-594 also colocalizes with rMAL-GFP. No ETX-594 binding was observed in the presence of JL004. JL001.2, JL002, and JL008 inhibited ETX internalization, but still allowed ETX-594 binding to cells and colocalization with plasma membrane-associated rMAL-GFP (white asterisks). (**C**) Western blot analysis for ETX oligomerization in rMAL-CHO cells via detection of the ~150 kDA ETX-oligomer complex. ETX was pretreated with indicated antibodies before treating cells. rMAL-CHO cells treated with ETX only was used as a positive control for ETX oligomerization. ETX oligomerization was detected by probing membranes with JL004, an antibody that has been determined to detect the approximately 150 kDa ETX-oligomer (Figure 1). (**D**) Full**-**length western blots of rMAL-CHO cells treated with ETX and ETX pretreated with indicated antibodies. Bands observed at ~50 kDa are believed to be the heavy chains of the anti-ETX antibodies used for pretreatment. (**E**) ETX-induced vacuolation and formation of Rab7 positive late endosomes were evaluated in rMAL-CHO cells. Cells were treated with ETX for indicated time points and stained for Rab7. Note at later time points, vacuole membranes stain positive for Rab7+ (white arrows). (**F**) JL001.1, JL001.2, JL004, and JL008 were evaluated for their ability to inhibit cellular vacuolation in ETX-treated rMAL-CHO cells via live cell imaging. Media containing ETX were pretreated with indicated antibodies prior to cell treatment.

To elucidate the mechanisms of action for the neutralizing ability of JL001.2, JL002, JL004, and JL008, a series of experiments was performed to determine if antibodies either blocked ETX binding or ETX oligomerization. To determine if antibodies could block ETX binding, media containing 50 nM of pETX fluorescently labeled with Alexa Fluor 647 (pETX-647) was pretreated with anti-ETX antibodies (15 μg/mL) for 30 min. Pretreated media was then incubated with rMAL-CHO cells for one hour and pETX binding analyzed by flow cytometry. Medium containing pETX-647 only was used as a positive control while media alone was used as a negative control. Only JL004 was able to inhibit pETX-647 binding to rMAL-CHO cells (Figure 8A).

These results were confirmed using ICC and active toxin fluorescently labeled with Alexa Fluor 594 (ETX-594) (Figure 8B). Cells treated with ETX-594 exhibited ETX binding and internalization of both rMAL-CHO and ETX-594. ETX internalization is consistent with previously published results [15]. Pre-incubation with JL004 inhibited ETX-594 binding to rMAL-CHO cells. Interestingly, ETX-594 pretreated with JL001.2, JL002, and JL008 allowed ETX-594 binding to cells, but inhibited internalization of ETX-594 or rMAL-GFP. As ETX internalization is believed to be a result of ETX oligomerization and pore formation, this indicates that JL001.2, JL002, and JL008 may inhibit ETX oligomerization.

To further, investigate if JL001.2, JL002, and JL008 inhibit ETX oligomerization, the presence of the ETX-complex after ETX pretreatment with antibodies was examined by WB analysis (Figure 8C). Again, media containing ETX was pretreated with antibodies prior to rMAL-CHO treatment. Cells treated with ETX alone were used as a positive control. Pretreatment of ETX with JL001.1, JL005, and JL006 did not inhibit oligomerization. Pretreatment with JL001.2, JL002, JL004, and JL008 did inhibit oligomerization. Full-length WBs of toxin-treated cells as well as cells treated with pre-incubated ETX were examined to address if ETX monomer binding could still occur while in the absence of ETX oligomerization (Figure 8D). ETX pre-incubation with JL001.2, JL002, and JL008 allowed ETX monomer to bind to cells while inhibiting oligomerization, most noticeably at longer exposure times. This confirms flow cytometry results (Figure 8A). In wells pretreated with anti-ETX antibodies, a band of approximately 50 kDa was observed, and is believed to be the heavy chain of our monoclonal antibodies. The protein was recognized in these WBs because we use peroxidase-conjugated anti-rabbit antibody as our secondary antibody. Taken together, this data indicates that JL004 exhibits neutralizing effects on ETX by blocking ETX binding, while JL001.1, JL002, and JL008 exhibit neutralizing affects by blocking ETX oligomerization.

ETX oligomerization and pore formation has also been linked to vacuolation in Madin-Darby Canine Kidney (MDCK) cells, believed to be a result of endocytosis of ETX oligomers into EEA1 early endosomes and eventually Rab7-positive late endosomes and lysosomes [51]. Analysis of rMAL-CHO cells revealed vacuolation and changes in Rab7 staining (Figure 8E). Vacuolation was observed at 60 min post treatment but became most abundant 120 min post treatment. ETX treatment also caused a change in Rab7 staining intensity and localization. As early as 30 min post-ETX treatment and increase in Rab7 staining was observed. By two hours post-ETX treatment, the vacuole membranes of ETX-treated cells were Rab7-positive, confirming previous reports. To determine if our anti-ETX antibodies could inhibit ETX-induced vacuolation and therefore endocytosis of the ETX-complex, media containing 50 nM of ETX were pretreated with JL001.1, JL001.2, JL004, and JL008 prior to addition to cells (Figure 8F). Vacuolation of cells was determined by live-imaging microscopy and vacuolation was used as an indicator of endocytosis. Cells could not be stained for Rab7, as the anti-Rab7 antibody is a rabbit antibody. Incubation of rMAL-CHO cells lines resulted in massive vacuolation after four hours of treatment. Pretreatment of ETX with JL001.1 failed to inhibit vacuolation while pretreatment with JL001.2, JL004, and JL008 inhibited vacuolation. Taken together, this data indicates that inhibition of ETX oligomerization by these antibodies may be inhibiting endocytosis of the ETX-complex as well.

### 3.9. Post-Exposure Treatment With Anti-ETX Antibodies Protects Against Cytotoxicity in Vitro and in Vivo

**Figure 9:**
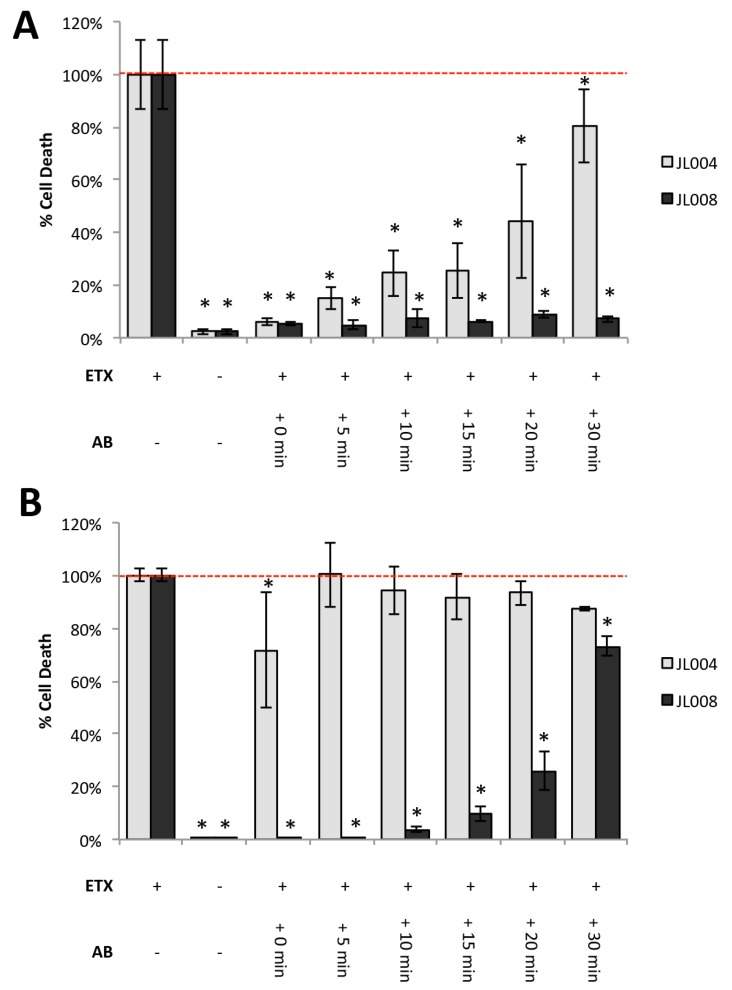
Post-exposure antibody treatment protects against ETX-induced cytotoxicity in vitro. rMAL-CHO cells were treated with 100 nM ETX for four (**A**) or 24 h (**B**). Cells were treated with ETX without antibodies as a positive control. Cells treated without ETX or antibodies were used as a negative control. Anti-ETX antibody JL004 or JL008 were added to cells treated with ETX post treatment at indicated time points; 0, 5, 10, 15, 20, or 30 min. Cell death was determined by PI exclusion. Percent cell death (% Cell Death) was determined by dividing the number of PI+ cells in experimental conditions by the number of PI+ cells when cells were treated with ETX alone. * *p* < 0.01 compared to ETX-treated controls determined by ANOVA with post-hoc Tukey HSD.

To determine if antibodies could protect from ETX-induced cytotoxicity after toxin exposure, in vitro experiments using rMAL-CHO cells were first evaluated (Figure 9). rMAL-CHO cells were treated with 100 nM ETX, followed by 30 μg/mL of JL004 or JL008, 0, 5, 10, 15, 20, and 30 min post-ETX treatment. 100 nM ETX treatment was chosen to maximize cell death. JL004 was chosen because it blocks ETX binding and JL008 was chosen because it blocks oligomerization. Cell death was evaluated four (Figure 9A) and 24 h (Figure 9B) after ETX treatment via PI inclusion assay. Four hours post-ETX treatment, JL004 and JL008 both significantly inhibited cell death at all post-exposure time points (Figure 9A). However, cells treated with JL004 had significantly more cell death than cells treated with JL008 at later time points. Differences in the protective effects of JL004 and JL008 were more apparent after 24 h of ETX treatment (Figure 9B). Cell death in cells treated with JL004 and ETX at the same time (0 min) was significantly inhibited compared to ETX-treated controls (71.6% vs. 100%, respectively). However, treatment with JL004 5, 10, 15, 20, and 30 min post-ETX treatment did not protect against ETX cytotoxicity after 24 h. In comparison, treatment with JL008 significantly inhibited ETX-induced cell death at all time points. When cells were treated with JL008 at 0, 5 and 15 min post-ETX treatment, ETX-induced cell death was below 5% of the ETX-treated controls. Treatment with JL008 at 15, 20 and 30 min post-ETX treatment reduced cell death to 9.6%, 25.9%, and 73.2% compared to ETX-treated controls, respectively, indicating JL008 is more protective than JL004 post-ETX exposure.

**Figure 10:**
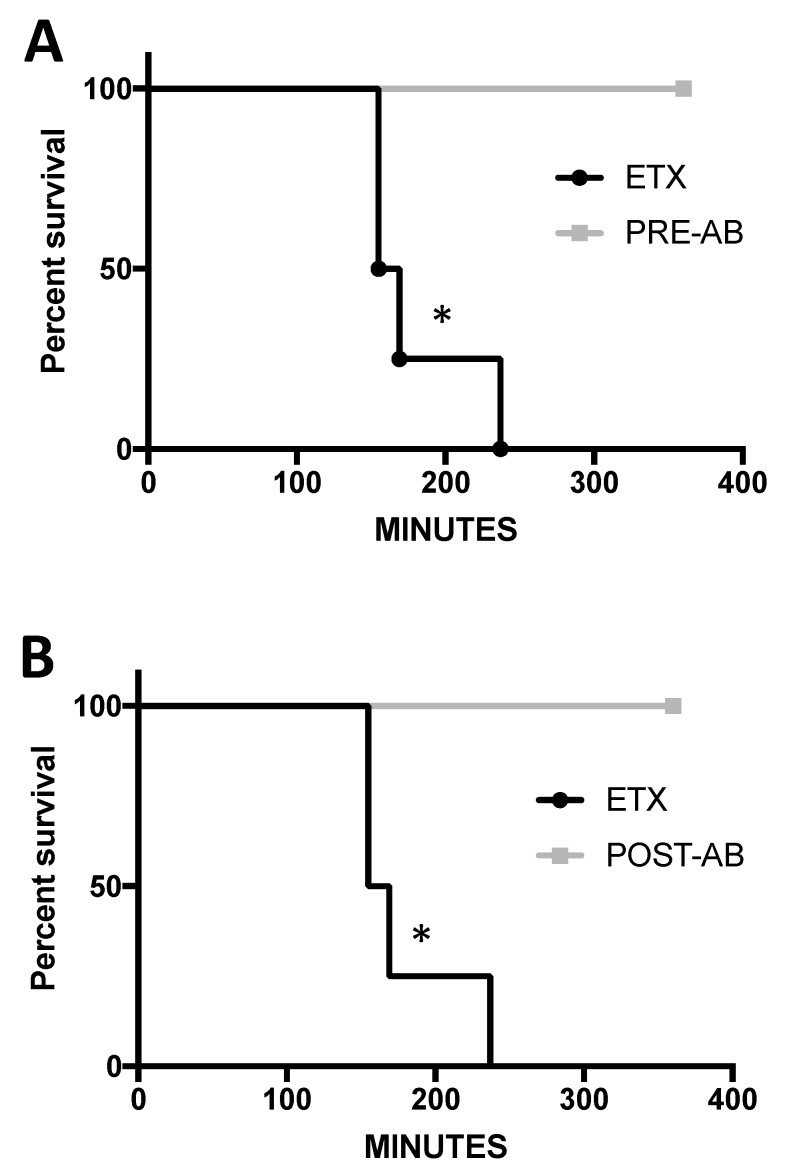
Anti-ETX antibody protects mice from ETX-induced central nervous system (CNS) symptoms in vivo. Mice were treated ETX via intraperitoneal injection. To determine if anti-ETX antibody JL008 could protect against ETX-induced CNS symptoms and death, mice were either treated with (**A**) JL008 30 min prior to ETX treatment (PRE-AB, *n* = 3) or (**B**) one hour post-treatment via intravenous injection (POST-AB, *n* = 3). Mice treated with ETX and intravenously injected with saline were used as controls (ETX, *n* = 4). Animal were observed for up to 360 min post-ETX treatment. Antibody treatment prior to and post-ETX treatment prevented ETX-induced CNS symptoms and death compared to ETX-treated control animals. The same control ETX group was used for both comparisons. * *p* < 0.02 determined by Log-Rank (Mantel-Cox) test.

Based on in vitro experiments, the ability of JL008 to protect mice from ETX-induced death and neurological changes were evaluated in vivo (Figure 10). Mice were treated with JL008 both before and after ETX treatment. 100 μg of JL008 were delivered intravenously either 30 min prior to or one hour after ETX treatment. Mice were treated with 5 ng of ETX per gram body weight via intraperitoneal injection. This dose was selected as preliminary experiments revealed that this treatment resulted in moribund symptoms within hours. ETX-treated mice injected intravenously with saline were used as controls. Mice were observed up to 360 min for ETX-induced moribund neurological symptoms (convulsions, extreme lethargy, shaking, loss of balance), and then humanely euthanized. Neurological symptoms observed at this time point are believed to be a result of ETX-induced blood brain barrier permeability, a well-accepted, but poorly understood process [52,53,54,55,56,57]. Both the pre- and post-treatment with JL008 protected mice against ETX-induced death. The mean time for symptoms in the ETX saline-treated mice was 179 ± 39 min. All mice treated with JL008 either pre- or post-ETX exposure survived the full 360 min and never exhibited neurological affects. This indicates that JL008 can protect mice from ETX-induced neurological effects and death pre and post-ETX exposure, under these conditions.

## 4. Discussion

In this paper, we describe the production of seven rabbit monoclonal antibodies cloned from B-cells of pETX-immunized rabbits. Although other authors have generated anti-ETX antibodies for use in ETX detection and neutralization [35,36,37,38,39,40,41,42], to the best of our knowledge, this is the first time a panel of monoclonal anti-ETX antibodies and their uses in various assays have been thoroughly described (Table 1). While all antibodies recognized purified ETX and pETX via WB, only JL001.2, JL004, JL005, and JL008 recognized bound ETX monomer on rMAL-CHO cells. Only JL004 and JL005 recognized the ETX-oligomer complex. In addition, JL002, JL004, and JL008 were best suited for ETX detection via ELISA, as JL005 produced excessive background signal. JL001.2, JL002, and JL008 were the only antibodies able to detect bound ETX on rMAL-CHO cells either via ICC or flow cytometry. Finally, we demonstrate that JL004 neutralizes ETX by blocking binding, while JL001.2, JL002, and JL008 neutralize ETX by blocking ETX oligomerization. Because of their diverse behaviors in various assays, these antibodies provide researchers with a valuable toolbox for studying ETX in a wide array of applications.

Previous efforts for detection of ETX in biological samples have focused on ETX-recognition using various ELISA and chromatography formats with varying levels of sensitivity [36,40,41,42]. In some ELISA formats, toxin levels could be detected at ranges from 0.1 ng/mL to 4 ng/mL, depending on the test buffer [40,41]. However, the LOD for these assays were not clearly determined. Interestingly, Féraudet-Tarisse et al. developed several different enzyme immunoassays and chromatography-based assays using anti-ETX mouse monoclonal antibodies with excellent sensitivity, establishing LOD ranging from 0.15 pM to 3.5 pM when evaluated in buffer [36], comparable to our LOD of 2.65 pM for our sandwich ELISA. Importantly, Féraudet-Tarisse et al. also tested their assays using an array of test matrices including milk, tap water, intestinal contents, and sera, and maintained LOD that far exceeded previously published methods.

Although these are important assays with excellent sensitivity, it is unclear if the antibodies selected for these assays would work in other formats such as ICC, immunohistochemistry (IHC), and flow cytometry. In our panel, only four antibodies worked for ELISA, and of these, only two worked for ICC or flow cytometry. Using monoclonal antibodies with high sensitivity offers great advantage over using polyclonal antibodies that often have high background issues and non-specific binding. Similarly, affinity-purified polyclonal antibodies can vary from prep to prep or lot to lot, leading to inconsistent results and requiring additional time and money to determine the proper working concentration. There is also the argument that rabbit monoclonal antibodies have higher antigen affinity and more robust results in various assays compared to mouse monoclonal antibodies [43,44,45,46]. Having high affinity monoclonal antibodies that recognize ETX in biological tissue or cells, such as JL001.2, JL002, and JL008, will aid in studying ETX-mediated diseases such as enterotoxaemia in ruminants and possibly MS in humans. Importantly, having a range of reagents including antibodies from different sources such as those described in previous publications is a benefit to researchers studying the mechanisms of ETX cytotoxicity.

Three of our monoclonals including JL001.2, JL002, and JL008 blocked ETX oligomerization. This was determined by showing that pre-incubation of ETX with JL001.2, JL002, or JL008 had no effect on binding of ETX to rMAL-CHO cells by flow cytometry and ICC, but did prevent activation of endocytosis, a characteristic cellular event induced by ETX oligomerization/pore formation [15,51]. Furthermore, lysates of cells treated under these conditions revealed bound monomeric ETX but not pore complex by WB. Since JL001.2, JL002, and JL008 do not detect the heat and SDS-resistant oligomer complex on WB, the epitopes recognized by these antibodies are likely masked in the ETX oligomeric complex.

The direct observation via WB that JL001.2, JL002, and JL008 inhibit ETX oligomerization is a novel finding. While other anti-ETX-neutralizing antibodies have been presumed to block ETX oligomerization or pore insertion into the membrane [35,36,37,38,39], to the best of our knowledge, this is the first time inhibition of ETX oligomerization has actually been directly visualized via WB analysis. Previous publications, including those with the most well studied anti-ETX-neutralizing antibody, 4D7, have used epitope mapping or indirect experimental evidence such as exclusion of a non-permeable cell dye to conclude that their antibodies block oligomerization or pore insertion. We agree with these authors’ conclusions and suggest that using JL004 to visualize oligomerization via WB could validate their assumptions. 

Because most of the JL anti-ETX antibodies perform differently in various applications, it seems plausible that they recognize separate ETX epitopes. It is difficult to predict which epitopes the non-neutralizing antibodies recognize. By using overlapping ETX peptides, Alves et al. were able to determine 15 epitopes that are recognized in ETX hyperimmune rabbit serum and identified four epitopes that appear to be immunodominant [34]. Interestingly, the most immunodominant peptide was (^116^TTTHTVGTSIQATAKFTVPFN^136^). The authors attributed this immunodominance to high hydrophobicity and the “exceptional accessibility” on ETX’s 3D structure. It is tempting to hypothesize that some of our non-neutralizing antibodies may recognize this specific domain.

Because we were able to determine the mechanisms of action for our neutralizing antibodies (JL001.2, JL002, JL004, and JL008) we can better hypothesize which domains these antibodies recognize. Because JL004 was able to block ETX binding, it is probable that this antibody recognizes an epitope in ETX domain I, the domain responsible for ETX binding to its receptors [23,24,25,26,27,28,29,30,31,32,33]. Because JL001.2, JL002, and JL008 block ETX oligomerization and possibly pore formation, it is likely that these antibodies recognize epitopes in domain II or III, which are believed to be important in ETX oligomerization and pore insertion.

Importantly, we demonstrated that JL008 was able to protect against ETX toxicity both in vitro and in vivo, post-ETX exposure. In vivo administration of JL008 one-hour post-ETX treatment completely protected mice from ETX-induced CNS dysfunction and death, believed to be a result of overt ETX-induced blood brain barrier permeability. Based on these observations, the question arises, how does JL008 inhibit ETX-induced cell death post-ETX-exposure? When soluble ETX is pretreated with JL008, we observe inhibition of cell death (Figure 7) as well as ETX oligomerization, but not binding to rMAL-CHO cells (Figure 8A,B). Under these conditions, we believe that JL008 is binding to soluble ETX, completely preventing oligomerization, most likely by blocking critical amino acid residues in domain II or III as previously discussed. However, JL008 also inhibits ETX-induced cell death post-ETX exposure (Figure 9), after ETX oligomerization has already occurred (Figure 2). In Figure 9, rMAL-CHO cells are exposed to 100 nM of ETX and then treated with JL008 0, 5, 10, 15, and 30 min post-ETX treatment. Notably, addition of JL008 at all time points significantly inhibited cell death 4 and 24 h after ETX treatment. However, ETX oligomerization (Figure 2) happens within 5 min after 50 nM ETX treatment and increases with time. Based on this data, JL008 may be inhibiting cell death post-ETX exposure using several different mechanisms.

To better address this, we first need to determine the steps essential for ETX cytotoxicity. These include (1) the presence of soluble toxin in medium, (2) initial monomer binding to receptor, (3) critical density of ETX monomer bound to receptors to allow for intermolecular interactions favoring ETX oligomerization, (4) insertion of formed ETX oligomers into the cell membrane to generate a functional pore, and (5) a sufficient number of functional pores to cause cell death. This last step is a kinetic balance of pore formation and removal of pores via endocytosis, a well-documented repair mechanism for pore-forming toxins [58,59,60,61,62] and a process observed in ETX-treated cells [15,51].

JL008 may be inhibiting cell death post-ETX exposure at early time points by either binding to unbound ETX in medium, therefore preventing ETX oligomerization and/or binding to bound ETX monomer on the cell surface, again preventing ETX oligomerization. Inhibition of additional ETX oligomerization could prevent formation of the critical number of ETX pores needed to achieve cell death. In cells that already have functional pores, this inhibition may also allow cells time to repair the pores formed in their cell membrane by endocytosis, therefore preventing cell death. In addition, because ETX oligomerizes on the cell surface prior to insertion of a functional pore into the cell membrane, JL008 may inhibit ETX oligomers from penetrating the plasma membrane, inhibiting formation of a functional pore as similarly proposed for other anti-ETX-neutralizing antibodies [35]. Finally, JL008 may protect against cell death by disintegrating already formed ETX oligomers or pores. The exact mechanism of how JL008 and the other neutralizing antibodies inhibit ETX-induced cell death is an area of ongoing research.

Because of the speculative mechanism of how JL008 protects against cell death post-ETX exposure, JL008 effectiveness as a prophylactic treatment to ETX exposure would depend on the ETX dose, the time between ETX exposure and treatment, and the sensitivity of the cells or species exposed to ETX. It is known that CHO cell lines expressing human MAL (hMAL) are less sensitive to rMAL-CHO cells [15]. The protective effect of JL008 post-ETX exposure would likely be of most benefit to species with lowered sensitivity to ETX exposed to relatively lower levels of ETX. Nevertheless, JL008 and the other neutralizing antibodies could possibly be used to treat livestock afflicted with enterotoxaemia or protect humans against a biological terrorism attack with ETX. Finally, if ETX does cause new MS lesion generation in humans, humanization of the neutralizing antibodies may be an essential step toward preventing MS disease progression.

## 5. Conclusions

In conclusion, we present seven unique anti-ETX rabbit monoclonal antibodies and their use in various assays. We believe these antibodies will provide useful tools for understanding the mechanisms of ETX cytotoxicity as well as ETX-mediated diseases including enterotoxaemia and possibly MS.

## 6. Patents

The antibodies described in the publication are the subject of granted and pending patent applications.

## Figures and Tables

**Table 1:** Summary of antibody function in different assays.

Antibody	Western Blot				ELISA	Flow	ICC	Neutralizing	Neutralizing MOA
	pETX	ETX	Bound ETX	Pore	ETX	ETX	ETX		
JL001.1	+	+	−	−	−	−	−	−	n/a
JL001.2	+	+	+	−	+	+	+	+	Oligomerization
JL002	+	+	-	−	−	+	+	+	Oligomerization
JL004	+	+	+	+	+	−	−	+	Binding
JL005	+	+	+	+	+	−	−	−	n/a
JL006	+	+	-	−	−	−	−	−	n/a
JL008	+	+	+	−	+	+	+	+	Oligomerization

## Supplementary Materials

The following are available online at http://www.mdpi.com/2073-4468/7/4/37/s1, Figure S1: Gating strategy for detection of ETX on rMAL-CHO cells via flow cytometry, Figure S2: Representative flow cytometry histograms for ETX detection with additional controls.
