# Supplementary material for: A Novel Panel of Rabbit Monoclonal Antibodies and Their Diverse Applications Including Inhibition of Clostridium perfringens Epsilon Toxin Oligomerization
Source: Antibodies (Basel). 2018 Oct 25;7(4):37. doi: 10.3390/antib7040037 (PMC6698963; doi:10.3390/antib7040037)
Supplement: Supplementary file 1 [file antibodies-07-00037-s001.pdf]

antibodies

Supplementary: A Novel Panel of Rabbit Monoclonal Antibodies and Their Diverse Applications Including Inhibition of *Clostridium perfringens* Epsilon Toxin Oligomerization

Jennifer R Linden [1], Kiel Telesford [1], Samantha Shetty [1], Paige Winokour [1,2], Sylvia Haigh [1], Ellen Cahir-McFarland [3], Giovanna Antognetti [3], Abhishek Datta [3], Tao Wang [3], Werner Meier [3] and Timothy Vartanian [1,*]

1 Brain and Mind Research Institute, Weill Cornell Medical College of Cornell University, New York, NY, 10065, USA; jel2049@med.cornell.edu (J.R.L.); kit2003@med.cornell.edu (K.T.); samanthashetty25@gmail.com (S.S.); pwinokur@mail.rockefeller.edu (P.W.); sylviavhaigh@gmail.com (S.H.)
2 Rockefeller University, New York, NY, USA, 10065
3 Biogen, Cambridge, MA, 02142, USA; ellen.cahir-mcfarland@biogen.com (E.C.-M.); giovanna.antognetti@biogen.com (G.A.); dradatta2004@gmail.com (A.D.); wt76610@gmail.com (T.W.); werner.meier@biogenidec.com (W.M.)
* Correspondence: tiv2002@med.cornell.edu

Received: 28 August 2018; Accepted: 22 October 2018; Published: 25 October 2018

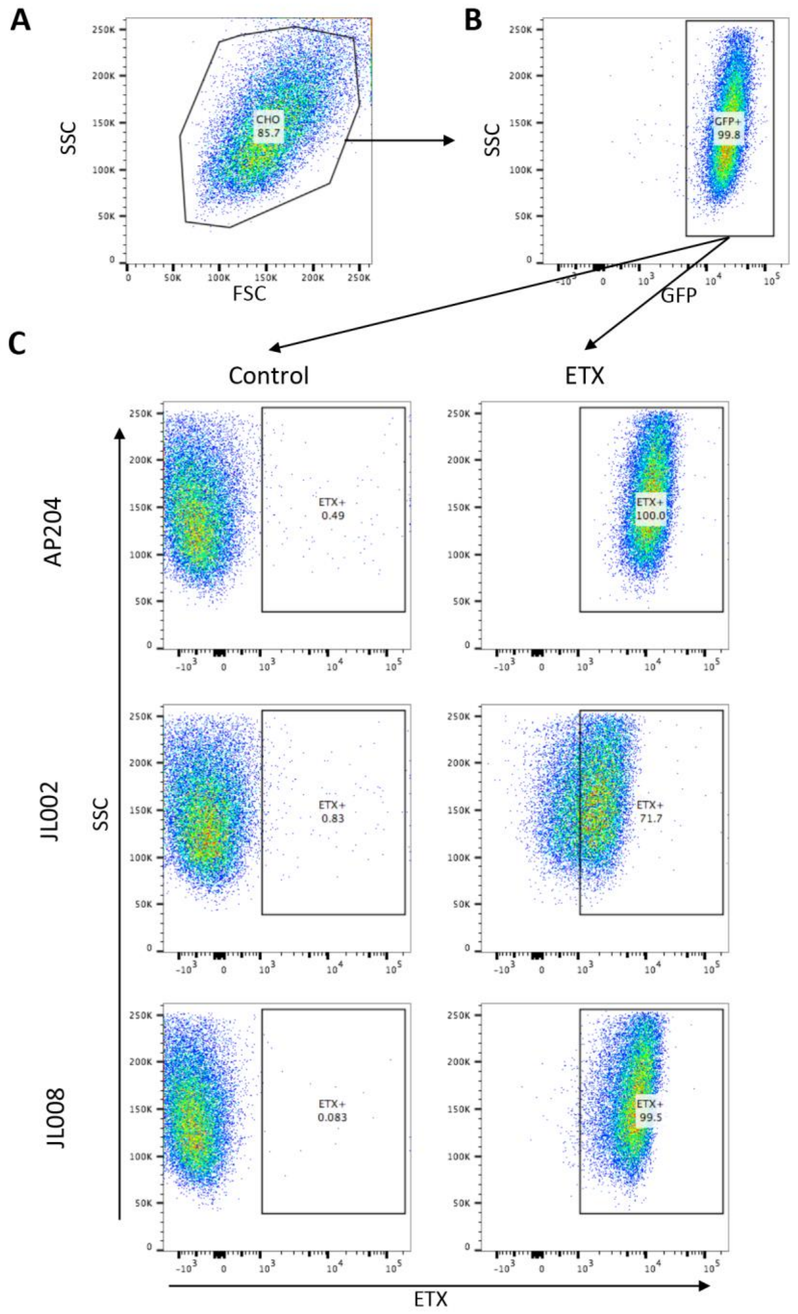

Supplemental Figure 1. Gating strategy for detection of ETX on rMAL-CHO cells via flow cytometry. rMAL-CHO cells were treated with (ETX) or without ETX (CT) for 1 hour. Cells were trypsinized, fixed, blocked then probed with antibodies at XXX for 30 minutes. Antibody binding was detected using an PE conjugated anti-rabbit antibody. **(A)** CHO cells were gated on based on the forward and side scatter. **(B)** rMAL-GFP expressing cells were gated on based on GFP fluorescence. **(C)** Example of anti-ETX antibody detection on control treated cells or ETX treated cells when probed with affinity purified anti-ETX polyclonal antibody (AP204), JL002, and JL008. Representative examples of experimental triplicates. Note, dot blots for JL008 are the same displayed in Figure 6B.

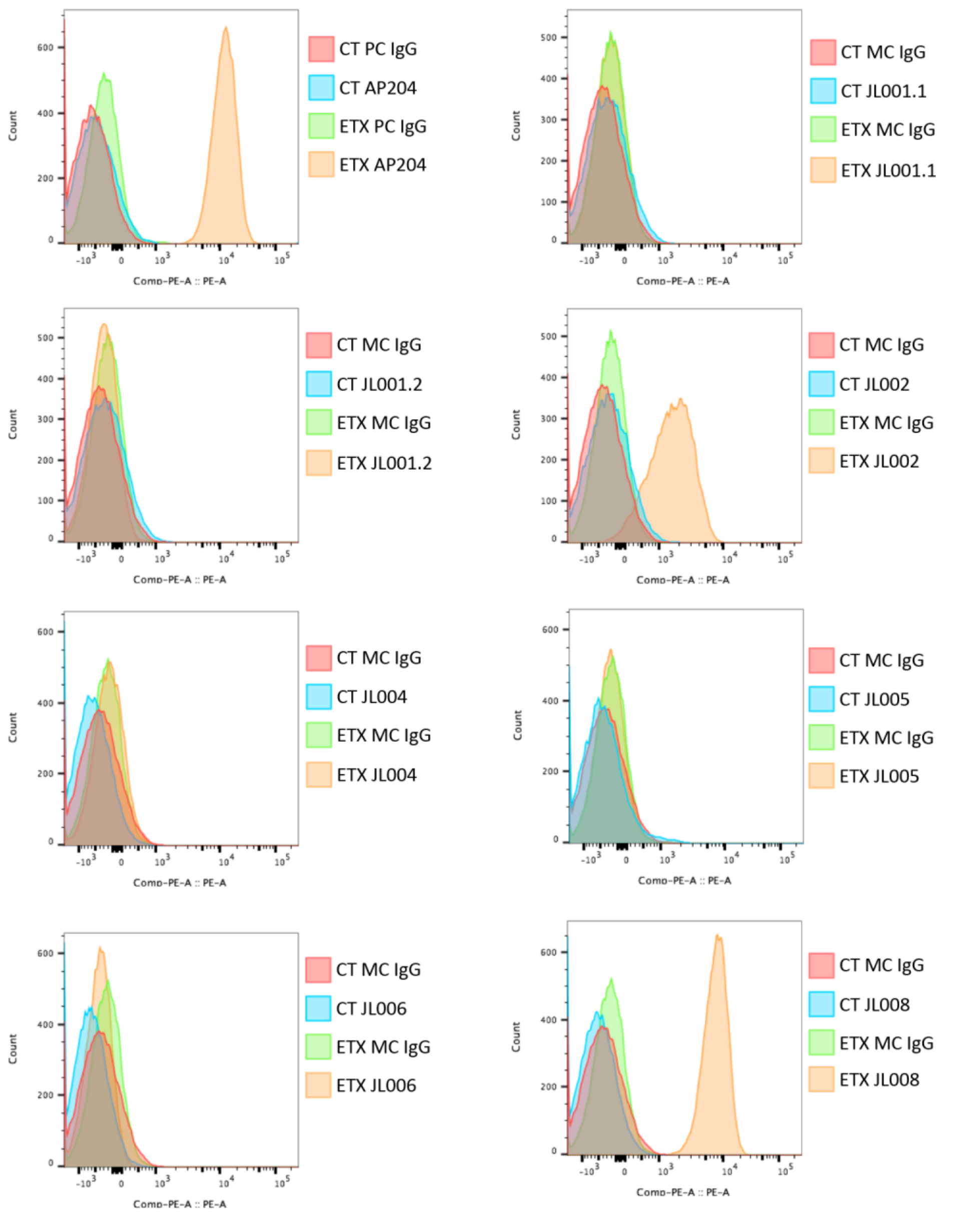

Supplemental Figure 2. Representative flow cytometry histograms for ETX detection with additional controls. Histograms of ETX florescence for control treated (CT) and ETX treated (ETX) rMAL-CHO cells when probed with individual anti-ETX monoclonal antibodies or AP204 polyclonal antibody. As additional controls, CT and ETX treated cells were also probed with appropriate isotype controls, a polyclonal IgG (PC IgG) for AP204 and a monoclonal IgG (MC IgG) for JL antibodies.
